# Opportunities in the translational pipeline for pediatric brain cancer therapies

**DOI:** 10.1038/s41390-025-03847-y

**Published:** 2025-02-01

**Authors:** Lindsay Rumberger Rivera, Nora L. Springer, Katherine Bailey, Jenny Patel, Christopher Brett, Elizabeth Barker

**Affiliations:** 1 University of Tennessee Graduate School of Medicine, Knoxville, TN USA; 2 Department of Biomedical and Diagnostic Sciences, University of Tennessee College of Veterinary Medicine, Knoxville, TN USA; 3 Department of Mechanical, Aerospace, and Biomedical Engineering, The University of Tennessee at Knoxville, Knoxville, TN USA

## Abstract

**Abstract:**

Primary malignant central nervous system (CNS) tumors are the leading cause of cancer-related mortality in the pediatric population. Moreover, survivors often experience significant long-term treatment-related morbidity. Challenges unique to drug delivery to the central nervous system have hampered therapeutic progress. In the past decade, significant advancements in our understanding of molecular biology, genetic alterations, and the tumor microenvironment have allowed us to improve our in vitro and laboratory animal models to better replicate diseases seen in the pediatric population. Recently, a comparative approach using naturally-occurring CNS malignancies in dogs with similar disease progression, histologic presentation, and treatment response has been proposed as an enticing model system. Given these improvements in the translational pipeline, there is an opportunity to identify and implement effective therapies more efficiently to pediatric CNS malignancy populations.

**Impact:**

- Relevant and translational pre-clinical studies are needed to find chemotherapeutics and targeted agents that can reach therapeutic doses within tumors in children without causing systemic adverse effects.
- A discussion of comparative oncology is provided with the intent to foster veterinary/human oncology collaboration.
- While the traditional pipeline for translating medications from bench to bedside has been evolving and improving over the last decade, the advances and remaining roadblocks of this pipeline are reviewed and discussed in this article.

## Introduction

Pediatric brain and other central nervous system tumors are the leading cause of cancer-related fatalities in children and adolescents aged birth to 19 years old.^1,2^ Improvements in mortality in pediatric and adolescent primary central nervous system (CNS) malignancies have lagged behind that of other cancers.^3^ Presenting further challenges to this population, survivors experience significant life-long morbidity from treatment. They have a higher incidence of chronic health conditions including pulmonary fibrosis, cardiac dysfunction, endocrinopathies, neuropathies, and neurocognitive deficits.^4–12^ Generating novel therapeutic strategies to improve outcomes in morbidity and mortality is critical. Given the rarity of individual childhood malignancies, it is difficult to adequately power prospective clinical trials, which further hampers the rate at which prospective new treatments can be translated to the bedside.

Historically, the clinical translation pipeline focused on testing the safety and efficacy of various therapeutic modalities in adults and subsequently imposed those on pediatric populations. The Best Pharmaceuticals for Children Act (BPCA) was implemented in 2002 to incentivize the development of improved pediatric therapeutics through preclinical and clinical drug trials that lead to the development of dosage forms specifically for pediatric use. The BPCA and subsequently the Pediatric Research Equity Act (PREA) in 2003 were created to address the need to establish correct doses, indications, side effects and safety profiles specifically for drugs and drug products for children.^13–22^ Originally, the focus was to develop oral dosage forms for pediatric drug products, but since then the scope has been expanded to include the development of appropriate pediatric formulations and pediatric drug delivery systems.^21^ While there are significant FDA incentives for developing pediatric drug products, the pharmaceutical industry has still been slow to produce new medicines for children, leaving the responsibility of this work primarily to universities and non-profit research institutions. There is a need for more relevant pre-clinical studies to find chemotherapeutics and targeted agents that can reach therapeutic levels within tumors in children without causing systemic adverse effects. The pipeline for translating therapies from bench to bedside has been evolving and improving over the last decade. The advances and remaining roadblocks of this pipeline are reviewed and discussed in this article.

## The case for better preclinical models

Randomized clinical trials are the standard of care in establishing the safety, tolerability, and effectiveness of new agents; however, such investigations are incredibly cumbersome, both in terms of time and expense. One study estimates 4000 h of labor on average were required in the conduct of a 20 patient, one year, phase III trial, and a recent average cost for a phase III trial was $19 million.^23,24^ As such, there is a tremendous incentive to weed out those agents that will ultimately be found to be ineffective or poorly tolerated prior to progressing to human clinical trial. The “Holy Grail” of preclinical models then is the ability to quickly and inexpensively identify agents that will ultimately be found effective against disease with tolerable toxicity in actual patients. The variety of preclinical models currently in practice all address a subset of these objectives. The following sections discuss some of the factors that should be taken into consideration in designing a panel of preclinical studies.

## Tumor biology

Pediatric brain tumors have distinctly different characteristics and challenges from adult malignancies, specifically in tumor immunology, molecular profiles, and treatment response. These differences become even more challenging to understand, given that the nervous system continues to develop during the first decade of life. Major components to consider when developing treatment options are the tumor microenvironment, the blood-brain barrier, genetic components associated with pediatric malignancies, and the immature state of the immune system.^25^

Recent advancements in whole-genome and epigenetic sequencing have transformed our understanding of key biological differences between pediatric and adult brain tumors, supporting the idea that treatment strategies that are effective in adults cannot be generalizable to pediatric populations.^26–30^ Differences in the tumor microenvironment pose additional factors that limit drug penetration, such as elevated interstitial fluid pressure, hypoxia, acidity, confinement, and nutrient-deprived environments.^31–35^ Variations in the pediatric blood-brain barrier and other pediatric-specific pharmacokinetic variables further separate the adult and pediatric populations.^36–38^

Accurately recreating the pediatric brain tumor microenvironment in an experimental model is a complex and multilayered challenge. Within this milieu there are immune cells (including macrophages, T and B lymphocytes, dendritic cells, neutrophils, and natural killer cells), as well as stromal cells (including fibroblasts, mesenchymal stromal cells, and pericytes), blood and lymphatic vessels, the extracellular matrix (containing neurons, astrocytes, microglia, and oligodendrocytes), and signaling molecules (including cytokines, chemokines, and growth factors), all of which have influence on tumor behavior.^39,40^

Common molecular characteristics also often differ in pediatric tumors. Compared with adult brain tumors, pediatric tumors are associated with a lower total mutation burden but a higher incidence of epigenetic changes. As we better understand the import of these genetic, cytogenetic, and epigenetic alterations on the biology of disease, they are being progressively incorporated in the pathologic classification of both adult and pediatric malignancies.^40^

The blood-brain barrier presents a significant challenge to the delivery of any potential CNS therapeutic. It is comprised of a tight network of endothelial cells, astrocytes, pericytes, and microglia, protecting the CNS from pathogens as well as preventing neurotoxicity by inflammation. Nearly 98% of small molecules and 100% of large molecules do not cross the blood-brain barrier (BBB). However, different CNS tumors and even different subtypes of the same tumor can affect the permeability of the BBB in different ways, which may explain why some tumors are more susceptible to systemic chemotherapy. This is well illustrated by medulloblastoma, the most common malignant brain tumor in children. By WHO classification, medulloblastoma is divided into four distinct subcategories based on distinct histopathologic and molecular characteristics: WNT group, Sonic Hedgehog Group (SHH), group 3 characterized by aberrant Myc expression, and group 4 characterized by MYCN and CDK6 amplification. WNT pathway tumors appear to block blood-brain barrier formation through endothelial WNT signaling, whereas SHH pathway tumors exhibit a normal vascular phenotype.^41–43^

Additionally, the tumors themselves possess characteristics that limit the efficacy of chemotherapeutics. Structural, non-cancerous components, such as the extracellular matrix, as well as aberrant tumor vasculature, contribute to mechanical parameters that can limit drug penetration. These parameters include interstitial fluid pressure (IFP), acidity, hypoxia, and nutrient-deprived environments. Intravenously administered drugs relying on diffusion to reach the tumor matrix are thwarted by the IFP in tumors reaching microvascular pressures. This can lead to hydrostatic equilibrium or even a higher interstitial pressure within the tumor microenvironment.^44,45^

## Patient-derived cancer models

One of the challenges to progress in pre-clinical studies of pediatric brain cancer has been the limited availability of pediatric cell lines. Within the last decade there has been a resurgence of tissue sampling to improve designs for clinical trials and to increase our knowledge of tumor biology and genetics.^46^ The increased availability of tumor specimens, along with increased utilization of tissue banks allows more opportunities to develop pre-clinical ex-situ studies adapted to the unique biology of pediatric brain cancers.

There are several patient-derived cancer models—cell lines, patient-derived organoids (PDOs), patient-derived explants (PDEs), and patient-derived xenografts (PDXs)—that can drive a more personalized approach to treatment. Although more complex than traditional cell cultures, neurospheres may better represent tumor heterogeneity in vitro due to their ability to maintain cells engaged in differentiation. However, they cannot mimic the full complex tumor microenvironment.^47^ Combining these models with functional diagnostic platforms may succeed in successfully developing precision medicines where static histopathological and molecular measurement classifications of tumors have failed.^48^

Although often appearing similar when examined by light microscopy, individual pediatric diffuse high-grade gliomas comprise several biologically distinct subtypes based on characteristic molecular genetic alterations. Similarly, chemosensitivity varies among different pediatric glioma subtypes. For instance, a chemosensitivity analysis of pediatric glioblastoma, anaplastic astrocytoma, and other high-grade gliomas showed limited sensitivities to conventional chemotherapeutic drugs, including carboplatin, etoposide, irinotecan, temozolomide, and vincristine.^49^ Studies must be designed to encompass individual glioma subtypes given the heterogeneity in pathology.

One of the pediatric brain tumors in which such progress is sorely needed is diffuse intrinsic pontine glioma (DIPG), which has a five-year overall survival rate of less than 1%.^50,51^ To that end, patient-derived DIPG cell cultures and orthotopic xenograft models have recently been made available for further research and investigations into targeted treatments, and is already beginning to bear fruit.^52–54^ The Children’s Oncology Group Preclinical Consortium performed a cell culture analysis with 14 DIPG models to identify effective therapeutic strategies.^54^ This chemical screening resulted in three diffuse intrinsic pontine glioma cultures sensitive to only 14 of the 83 chemotherapeutic agents. Notably, this study demonstrated significant cell sensitivity and potency for histone deacetylase inhibitors.^54^ Whereas histone mutations are rare in adult high-grade gliomas, abnormalities in histone deacetylase activity are often present in pediatric high-grade gliomas and contribute to tumor proliferation and survival.^29,55–58^ In this study, Panobinostat, a multi-histone deacetylase inhibitor, was one of the most effective forms of therapy demonstrating a decrease in cell viability in diffuse intrinsic pontine glioma.^54,57^

This agent has recently transitioned to pediatric clinical trial, with a phase one dose-finding study completed in 2023. Although only an exploratory analysis, tumor effect at tolerated dosing has thus far been muted, with CNS penetration a potential limiting factor. While further investigation will remain required for this and other potential molecular targets identified moving forward, adapting research strategies to specifically target aberrant molecular pathways expressed in pediatric tumors remains critical for the development of novel agents.

Another case study highlighting the importance of genetic classification to obtaining generalizable data is the early misclassification of medulloblastoma cell lines. Most initial pre-clinical evaluation studies in the 1970s to 1980s were tested with the human cell line TE-671.^59,60^ TE-671 originated from a 6-year-old patient who was thought to have medulloblastoma, however the cell line was later shown to have mutations consistent with rhabdomyosarcoma.^61–63^ Medulloblastoma cell lines have since been classified by molecular subtype. Many cell lines, however, remain unclassified given substantial pathological heterogeneity. Further limiting applicability of studies, approximately 50% of citations of medulloblastoma cell lines are derived from DAOY, a Sonic Hedgehog Pathway (SHH) subtype cell line.^64^ The high utilization of a single cell line limits broad applicability of studies. More pre-clinical studies are needed to focus on drug distribution and chemosensitivity of the current standard of care treatments to determine the efficacy of chemotherapeutic drugs against alternative medulloblastoma tumor subtypes as well as other malignancies. With the increased knowledge of genetic and molecular profiling of the various pediatric brain tumors, in vitro studies can be conducted with improved cell lines for more streamlined translation.

## Animal models

The development of more comprehensive and genetically diverse cell line libraries is allowing progress in cell culture derived work; however, the tumor microenvironment has a critical bearing on the growth, development, and behavior of tumors, and in vivo models are required as candidate therapeutics are identified.

### Mammalian laboratory models

#### Tumor transplant murine models

The gold standard model for brain cancer research is the mouse model utilizing transplantation of cultured stem cells, patient-derived fresh tissue, isolated cell suspensions, or neurospheres into an immunodeficient mouse host.^47^ In its simplest form, this can often be achieved through the use of subcutaneous injection into a rodent model. This model recreates many of the factors that are lost in cell culture. However, for the study of pediatric brain cancer, a more authentic modeling of tumor microenvironment can be obtained through orthotopic tumor implantation directly into the model central nervous system. For example, the most common pediatric glioblastoma models are from orthotopic xenografting of patient tissue or patient-derived cell lines. These recapitulate both the histology and molecular landscape of the original patient tumor.^65^

The use of orthotopic rodent models, where a tumor is engrafted into the de novo location, and patient-derived tumor models, where samples are engrafted into animal models directly from patients, have allowed for improved pre-clinical therapy evaluations for multiple tumor types. They are becoming increasingly utilized as pediatric brain tumor models.^66–71^ A recent study used samples from 5 pediatric patient tumors including two ependymomas, two medulloblastomas, and one high-grade glioblastoma to generate murine orthotopic patient-derived xenograft models.^72^ This study demonstrated the benefits of an immunofluorescent protein, iRFP713, which allows for live intracranial monitoring without impacting tumor engraftment or growth when transfected.^72^ The use of cell lines and patient-derived samples transfected with the iRFP713 protein will allow for improved real-time imaging during pre-clinical studies utilizing orthotopic brain tumors, which would lead to improved monitoring of intracranial tumor burden. In addition to utilizing rodents to study tumor burden, small animals can also be utilized to study novel methods for delivering drugs past the blood-brain barrier. Some of these methods currently in pre-clinical studies focus on well-established cytotoxic medications, such as paclitaxel, by formulating the drug within ferritin heavy chain nanocages^73^ and serum albumin nanoparticles.^74^

An additional component of the tumor microenvironment that has gained increased recognition in recent years is the interaction between the tumor and the host immune system. One shortcoming of models based on transplantation of human-derived xenografts is their reliance on an immunodeficient host to permit implantation. An exciting possible solution to this problem is the development of “humanized” mouse models, in which a limited human-derived immune system is transplanted into the mouse prior to tumor engraftment.^75^ While these models bear their own difficulties, including the eventual development of graft versus host disease, the onset of this can currently be delayed as much as 20 weeks after engraftment, thus facilitating a window for effective study. Mice reconstituted with human bone marrow can be utilized to test immune therapies, however, these models have a high cost, and the ability of the mice to recapitulate human biology is still unknown.^40^

#### Genetically engineered mouse models

To study the early stages of tumor initiation in pediatric brain tumors, especially for understanding embryonal tumors that may arise from embryonic or fetal cells remaining in the CNS, a different type of mouse model is employed.^40^ Genetically engineered mouse models (GEMMs) seek to control target gene expression to better understand the tumor lifecycle, including (1) initiation and sustained tumorigenesis, (2) the clinical phenotype, (3) changes in signaling pathways, (4) secondary genomic alteration, and (5) potential therapeutic targets. This has been particularly successful in understanding the sequence of genetic events that underly neuroblastoma. Given that determination of an oncogenic gene in pediatric cancer relates to its role in development (as opposed to the accumulation of genetic mutations over time that is seen with adult malignancies), GEMMs can be particularly useful, because they combine elements of controlled and visual expression.^76^ GEMMs have even been used to study the BBB and its impact in different tumor formations and treatments.^47^

GEMMs are exceptional tools for determining how and where tumors might be expected to develop, but their applicability to preclinical drug development is not yet widely used.^77^ As opposed to orthotopic models, GEMMs have the benefit of naturally arising tumors within immunocompetent hosts, including all the native tumor-stromal interactions and tumor-derived vasculature. For example, both PDXs and GEMMs have been employed to study diffuse midline glioma, however direct implantation of patient-derived cells tends to lead to greater loss of cells in the transplantation process.^77^

GEMMs use is increasing in pediatric cancer research after successfully defining clinically important mutations in major adult cancers. However, a possible downside to these models in preclinical testing is that the tumor sensitivities to both traditional chemotherapies and novel small-molecule therapeutics may be exaggerated. They may also be cost and time prohibitive in a preclinical trial due to patents, long tumor development latencies, potential low tumor penetrance, and difficulties in monitoring tumor development along with therapeutic response.^76^

#### Organotypic brain slice culture

Organotypic brain slice culture (OBSC) platforms are living tissue substrates that are typically generated from rats or mice. In this model, a thin slice of intact brain tissue can be placed upon a semipermeable membrane atop the underlaying culture media, thereby maintaining a great cellular diversity and more authentic microenvironment than a traditional tissue culture. By engrafting uncultured patient brain tumor tissue on the OBSC substrate, the tumor growth, invasion, and cellular interactions can be observed. These substrates also allow for comparisons for normalized and direct drug-to-drug and tumor-to-tumor comparisons.^40^

Organotypic brain slice cultures combined with tumor spheroids are a useful way to evaluate the interactions between tumor and normal brain cells, because OBSCs preserve the cytoarchitecture of brain tissue and vascular cells while allowing for manipulation and observation directly within the culture dish. With this technique, specific brain regions can be targeted for study. Another advantage is that a single animal can generate multiple brain slices. This generates uniformity and reduces the number of required animals.^47^

A disadvantage to this model is that cultures are only viable for about 3 weeks, which makes them ill-suited for slower-growing malignancies and evaluating drug response. The lack of blood flow within the slices isolates the study from evaluating the effect of immune cells, and the slicing procedure itself results in microglial activation and triggers cell death.^47^

#### Pig models

There is no evidence of spontaneous glioma in pigs, however two glioma pig models have been developed from human cell-line xenografts (U87, U251, T98G, and AS172). The benefits of pig models are their stronger resemblance to human physiology versus rodents. However, these pig models must be immunocompromised to prevent immunological cross-reactivity with the human cells. This is typically accomplished through oral cyclosporine. Some limitations to this model include the immunocompromised host, which hinders the development of immunotherapy protocols, and the impact of the pig immune system on the xenograft cells has not yet been fully characterized. Another limitation is size. Common agricultural breeds weigh approximately 300 kg when mature, which is a challenge for most labs. Minipig models, with maximum size ranging from 35–90 kg, have been developed to overcome this issue.^78^

#### Non-human primates

Non-human primates are the most physiologically, anatomically, and genetically similar animals to humans, but their use as a preclinical model is limited by ethical considerations.^78^

## Non-mammalian laboratory models

There are two potential non-mammal models worth mentioning with regards to filling the translational gap in developing new therapies for pediatric brain and CNS tumors.

Drosophila Melanogaster presents an alternative to the glioma mouse model, because of a high percentage of functional orthologs between human genes and drosophila. While useful for studying tumor development, this model lacks an adaptive immune system, which limits its usefulness in the translation pipeline.^79^

Zebrafish models have been used to study glioblastoma by transplanting patient-derived tumors. The fish are optically transparent, allowing for direct imaging and visualization of both tumor cell behavior and host tissue interactions, although this transparency reduces with age. These models are less expensive than mice, and allow for direct study of the TME, which could make them extremely useful for large drug screening.^47^ The zebrafish develops its immune systems beyond 6-weeks of age, so early implantation of human glioma cells is particularly useful for studying tumor development. The microenvironment also has a similar density to the human brain. A major disadvantage to the model is that zebrafish cells prefer a temperature of 28 °C instead of the optimal 37 °C preferred by human cells.^79^ While fascinating, zebrafish models are not as robust as mouse models when considering organ systems, molecular pathways, and conservation of genes.^47^

## Comparative models: naturally-occurring CNS malignancies in companion dogs

In recent years, there has been a concerted effort to utilize naturally occurring cancers in companion animals, predominantly dogs, in the drug development pipeline to improve efficacy and time to market. Naturally occurring tumors in dogs have biological and clinical similarities to those that develop in people with the benefit of avoiding common pitfalls of traditional laboratory animal pre-clinical models such as immunosuppression, engineered genetic manipulations, and a sterile, controlled environment. Researchers have used canines with various tumor subtypes in pre-clinical and early clinical trials to investigate novel therapies that can be translated between the canine and human populations.^80–83^

Intracranial gliomas spontaneously arise in canines at frequencies similar to humans (ref ^75^). Spontaneous canine models provide natural tumor growth within a reasonable time period that responds to multi-step therapies similar to humans. Canine malignancies exhibit inter-patient heterogeneity (i.e. varying ages and breeds of dog; types and grades of glioma in different locations within the brain) and intra-patient heterogeneity (pleomorphic cells, necrosis, vascular proliferation, and pseudo-palisading) that are seen in human glioblastoma but not in murine xenografts.^84^

A Comparative Brain Tumor Consortium (CBTC) was developed by the Comparative Oncology Program of the National Cancer Institute to study naturally occurring canine gliomas to (1) find the similarities and differences between dog and human gliomas, and (2) create a classification scheme to help veterinarians consistently diagnose canine gliomas.^84^ This international consortium of physician and veterinary neuropathologists harmonized morphologic criteria for histopathological classification of gliomas and determined that canine malignant gliomas share phenotypic similarity to human pediatric gliomas.^84^ Using the updated histologic classification schema, a collaborative group analyzed the genetic patterns in 83 canine glioma tumor samples from 5 veterinary research hospitals and found aneuploidy, mutational rates, timing of mutations, and DNA-methylation patterns that were comparable to those found in human pediatric gliomas.^85^ Approximately 40% of canine gliomas carried at least one significantly mutated gene such as *TP53*, *PDGFRA*, *PIK3CA*, and *EGFR*, a similar proportion as pediatric gliomas.^86^ Additionally, despite mostly occurring in adult dogs, approximately 80% of canine gliomas would be classified as pediatric based on methylation pattern, being most similar to the H3 wildtype subtype.^85^ Collectively, these studies demonstrate similarities in phenotypic and genotypic tumor expression between canine and pediatric patients, suggesting canine models are an attractive translational model. Other advantages of the comparative dog model are practical: the canine nervous system is close in size to children, alleviating concerns over scalability and the limitations of cellular migration. The canine brain is also microscopically very similar to humans.^87^ Standard of care treatment of canine brain tumors is similar to humans, with surgical resection, radiation and chemotherapy options available.^88^

Translational success between humans and canines was demonstrated in a case study of a 6-year-old canine patient who presented with a midline medulloblastoma. Veterinarians used a treatment protocol adapted from pediatric medulloblastoma management. By utilizing a human pediatric medulloblastoma protocol for chemotherapy and surgery (radiation was declined by the owner), the canine patient had significant clinical improvement for months following his initial diagnosis.^89^ This and similar case studies illustrate the importance of utilizing canine models in the translational research pipeline.

One area where comparative models might be beneficial to both people and pets is targeted therapies. Numerous small-molecule inhibitors are available that target pathways known to be dysregulated in low and high-grade pediatric gliomas (Mueller 2020). Despite the ubiquity of targeted therapies in people, they are infrequently used in veterinary medicine due to limited approved drugs and expense. Veterinary clinical trials are held to high ethical standards but have regulatory flexibility compared to human clinical trials. This allows for more efficient testing of novel drug combinations, potentially benefiting people. On the other hand, access to therapies that are used off-label and cost-prohibitive could benefit companion animals and identify new markets to drug manufacturers.

Despite many potential advantages to canine cancer models, there are several obstacles to more widespread utilization. Although canine cancer progression is faster than in humans, is still much slower than murine models, further increasing the cost of large animal trials Other factors limiting the use of spontaneous canine models include: (1) cases may be diagnosed too late to benefit from treatment, (2) pet owners may be unable/unwilling to contribute financially to the trial, (3) ethical considerations surrounding repeated sampling, and (4) owners may end the trial early to prevent additional hardship on their pet and opt for humane euthanasia instead.^88^ Pet dogs cannot be seen as a phase 1 trial for new therapies. Prior in vitro and mice studies still need to be conducted before introducing novel therapies to the veterinary clinic.^88^

## From bench to bedside

Determining the amount of preclinical evidence required before a human clinical trial is allowed is a key hurdle in developing new diagnostic and treatment paths for CNS malignancies. Data collected from tumor bearing models can help predict what will occur in clinical human trials, however preclinical animal models cannot completely parallel the human disease progression or tumor response.^88^

While pre-clinical investigations will help us better understand the effectiveness of therapeutics in pediatric brain tumors, challenges in conducting the needed clinical trials for safety and efficacy persist. Inter-tumor heterogeneity within pediatric brain tumors narrows the ability to translate treatment regimens across tumor subtypes given their key biological and molecular differences. The international pediatric brain tumor community has been working for years to define genomic subtypes of pediatric brain tumors to allow for the development of tumor-specific therapy protocols.^90^

There is a deficiency of research in defining the barriers of the tumor microenvironment impeding drug penetration in pediatric brain tumors. Defining and developing methods to overcome and target these cellular, vascular, and immune barriers in the tumor microenvironment will advance the efficacy of therapies in treating pediatric brain tumors. Batista et al. presented methods to enhance pediatric brain cancer treatment by targeting the tumor microenvironment, including disrupting paracrine signaling between cancer cells, stromal cells, and matrix, and by targeting the tumor vasculature^91^ Snuderl et al. presented a study targeting the placental growth factor/neuropilin 1 pathway, inhibiting growth and spread of medulloblastoma.^92^ Tumor-stroma-targeted therapies are strategies that would disrupt signaling and physical interactions between cancer cells and their surroundings. This method could be advantageous because it would allow for therapies to target cells and lead to the identification of common targets among tumor subtypes, which could further lead to simplified and cost-effective clinical trials.^91^ However, little is known about the interactions of pediatric brain tumor cells with their microenvironment, which will need to be further characterized to allow for the discovery of new strategies.

These limitations further exemplify the need for continued pre-clinical studies so that better clinical trials can be developed. An international panel of clinicians and laboratory-based scientists gathered by Cancer Research UK presented key challenges to treating primary brain tumors. Among the challenges, they noted that clinical trials have not revealed effective therapies for most brain tumors and there is a need for international collaboration that transcends traditional research disciplines. International collaborations are key to developing better pediatric brain tumor therapies through comprehensive expertise in pre-clinical studies, clinical trials, and adaptive trial designs.^90^

Comparative oncology is the field of study that explores naturally occurring cancers in nonhuman species as complementary models for human cancer research. While no single animal can completely mimic human malignancy, models such as neurosphere cultures, patient-derived xenografts, and genetically engineered mice have been highly informative.^84^ Patient derived xenograft models provide great insight into the genetic and molecular investigation of malignancies such as medulloblastoma, however their reliance on immunocompromised hosts means they cannot investigate the immune system response as a whole. Immune-competent models could complement patient-derived xenograft models as part of the preclinical pipeline to optimize radiation, chemotherapy, and even immunotherapy protocols.^93^

Canine trials can provide valuable information to human cancer research due to clinical, biologic, molecular, and genomic similarities in tumors between the species, especially when coupled with robust murine models.^88^ An advantage of spontaneous tumor formation in pet dogs is the observation of the tumor within a natural microenvironment with intact immune system and BBB.^84^ Partnering with veterinarians for preclinical treatment of pet dogs can be beneficial to both fields. Owners of pet dogs are highly motivated to help their pets, and may be interested in participating in trials when standard-of-care options fail to meet their goals.^88^

However, overcoming the silos of human and veterinary medicine will be critical for the success of comparative medicine approaches. The infrastructure exists for robust multi-institutional veterinary clinical trials through the Comparative Oncology Trials Consortium^94^, a network of academic comparative oncology centers under the purview of the NIH-NCI-Center for Cancer Research Comparative Oncology Program. Comparative medicine symposia at academic institutions with both medical and veterinary colleges can foster interdisciplinary collaborations at the local level. Attending or speaking at conferences or publishing in journals from other disciplines can also raise awareness for the growing field of comparative medicine.

## Discussion

Current medical care for pediatric brain cancer struggles to rid patients of the disease without burdening them with lifelong health challenges. Early studies developing therapies for pediatric brain cancer relied heavily on adult clinical trials and pre-clinical models, all of which failed to fully recapitulate the molecular biology, genetics, and tumor microenvironment of pediatric brain tumors. This failure hindered the ability to translate effective therapies with minimized side effects. In more recent years, the increased profiling and generation of cell lines has improved the translatability of in vitro studies and has allowed for the use of orthotopic patient-derived murine tumor models for early in vivo investigations. While these improvements allow for more relevant early studies, large animal and early clinical studies are still needed.

Canine patients offer a truly unique opportunity given that their de novo tumors have biologically similar presentation and therapy responses. By utilizing the more robust and relevant in vitro, murine models, and veterinary canine patients alongside more collaborative international pediatric clinical trials, the bench-to-bedside therapy translation can be more efficient and effective at getting much-needed therapies to pediatric patients with brain tumors.
